# Supplementary material for: A Systematic Review and Meta-Analysis of Malignant Rhabdoid and Small Cell Undifferentiated Liver Tumors: A Rational for a Uniform Classification
Source: Cancers (Basel). 2022 Jan 6;14(2):272. doi: 10.3390/cancers14020272 (PMC8774069; doi:10.3390/cancers14020272)
Supplement: Supplementary file 1 [file cancers-14-00272-s001.zip › File S1.pdf]

**Search Strategy for the Systematic Review on MRTL and SCUD:**

Medline via Pubmed

((rhabdoid[tiab] OR (small cell undifferentiated[tiab] OR small cell variant[tiab] OR small cell tumor[tiab]) OR SCU[tiab] OR SCUD[tiab]) AND (liver[tiab] OR hepatic[tiab] OR hepatoblastoma[tiab])) OR ((Liver Neoplasms[MeSH] OR Hepatoblastoma[MeSH]) AND Rhabdoid Tumor[MeSH])

Web of Science

TS = ((rhabdoid OR "small cell undifferentiated" OR "small cell variant" OR SCU) AND (Liver OR hepatic OR Hepatoblastoma))

Central

(malignant rhabdoid):ti,ab,kw OR (small cell OR SCUD OR SCU):ti,ab,kw AND (liver OR hepatic):ti,ab,kw
