# Supplementary material for: A Systematic Review and Meta-Analysis of Malignant Rhabdoid and Small Cell Undifferentiated Liver Tumors: A Rational for a Uniform Classification
Source: Cancers (Basel). 2022 Jan 6;14(2):272. doi: 10.3390/cancers14020272 (PMC8774069; doi:10.3390/cancers14020272)
Supplement: Supplementary file 1 [file cancers-14-00272-s001.zip › File S2.pdf]

Supporting information File S2: List of included studies

Authors	Title	Year of publication	No° of Cases	Country
Gonzalez-Crussi et al.	*Infantile Sarcoma with Intracytoplasmic Filamentous Inclusions*	1982	1	USA
Parham et al.	*Malignant Rhabdoid Tumor of the Liver*	1988	2	USA
Pierro et al.	*Preoperative Chemotherapy in 'Unresectable' Hepatoblastoma*	1989	2	Canada
Hunt et al.	*Malignant Rhabdoid Tumor of the Liver A Distinct Clinicopathologic Entity*	1990	1	USA
Foschini et al.	*Malignant rhabdoid tumour of the liver. A case report*	1992	1	Belgium
Hansen et al.	*Undifferentiated Small Cell Hepatoblastoma with a Unique Chromosomal Translocation: A Case Report*	1992	1	USA
DiCori et al.	*Primary malignant rhabdoid tumour of the liver*	1993	1	Canada
Gururangan et al.	*Primary Extracranial Rhabdoid Tumors Clinicopathologic Features and Response to Ifosfamide*	1993	3	USA
Weymann et al.	*Secretion of Vasointestinal Peptide by a Primary liver Tumor With Rhabdoid Features*	1993	1	USA
Kaiserling et al.	*Immunohistochemical and cytogenetic findings in malignant rhabdoid tumor*	1996	1	Germany
Scheimberg et al.	*Primary Hepatic Malignant Tumor with Rhabdoid Features*	1996	3	UK
Tsunoda et al.	*NON-ALPHA-FETOPROTEIN-PRODUCING ANAPLASTIC HEPATOBLASTOMA CELL LINE*	1996	1	Japan
Pogacnik et al.	*Malignant Rhabdoid Tumor of the Liver Diagnosed by Fine Needle Aspiration Cytology A Case Report*	1997	1	Slovenia
Jimenez et al.	*Primary Hepatic Malignant Tumor With Rhabdoid Features*	1998	1	Spain
Kelly et al.	*Spontaneous rupture of a malignant rhabdoid tumour of the liver*	1998	1	UK
White et al.	*Congenital Disseminated Malignant Rhabdoid Tumor*	1999	1	USA
Donner et al.	*Translocation (8;13)(q24.2;q33) in a Malignant Rhabdoid Tumor of the Liver*	2000	1	USA
Ohyama et al.	*CONGEN!TAL PRIMITIVE EPITHELIAL TUMOR OF THE LIVER SHOWING FOCAL RHABDOID FEATURES, PLACENTAL INVOLVEMENT, AND CLINICAL FEATURES MIMICKING MULTIFOCAL HEMANGIOMA OR STAGE 4S NEUROBLASTOMA*	2000	1	Japan
Sattler et al.	*Undifferenziertes kleinzelliges Hepatoblastom*	2000	1	Germany
Haas et al.	*Small Cell Undifferentiated Histology in Hepatoblastoma May Be Unfavorable*	2001	16	USA
Ravindra et al.	*Long-Term Survival After Spontaneous Rupture of a Malignant Rhabdoid Tumor of the Liver*	2002	1	UK
Weir et al.	*Hepatoblastoma - Cytomorphologic Characteristics in Serous Cavity Fluids*	2002	1	USA
Katzenstein et al.	*Metastatic Malignant Rhabdoid Tumor of the Liver Treated With Tandem High-Dose Therapy and Autologous Peripheral Blood Stem Cell Rescue*	2003		USA
Yuri et al.	*Malignant rhabdoid tumor of the liver: Case report and literature review*	2004	1	Japan
Bajpai et al.	*Midterm results with hepatectomy after preoperative chemotherapy in hepatoblastoma*	2005	3	India
Kuroda et al.	*Establishment of a cell line from a malignant rhabdoid tumor of the liver lacking the function of two tumor suppressor genes, hSNF5/INI1 and p16*	2005	1	Japan
Clairotte et al.	*Tumeur rhabdoïde hépatique maligne avec rupture spontanée*	2006	1	France
Philipose et al.	*Cytologic Diagnosis of Small Cell Anaplastic Hepatoblastoma*	2006	1	India
Jayaram et al.	*Successful Management of Rhabdoid Tumor of the Liver*	2007	1	USA
Madigan et al.	*Extracranial Malignant Rhabdoid Tumors in Childhood*	2007	1	USA
Wagner et al.	*Malignant Rhabdoid Tumor Mimicking*	2007	1	USA

	Hepatoblastoma: A Case Report and Literature Review			
Bourdeaut et al.	*Extra-Renal Non-Cerebral Rhabdoid Tumours*	2008	2	France
De Ioris et al.	*Hepatoblastoma with a low serum alpha-fetoprotein level at diagnosis: The SIOPEL group experience*	2008	8	France
Gutweiler et al.	*Hepatoblastoma presenting with focal nodular hyperplasia after treatment of neuroblastoma*	2008	1	USA
Wu et al.	*Rhabdoid tumour: a malignancy of early childhood with variable primary site, histology and clinical behaviour*	2008	2	Australia
Abe et al.	*Malignant rhabdoid tumor of the liver: a case report with US and CT manifestation*	2009	1	Japan
Marzano et al.	*Malignant Rhabdoid Tumour of the Liver in the Young Adult: Report of First Two Cases*	2009	1	France
Trobaugh-Lotario et al.	*Small Cell Undifferentiated Variant of Hepatoblastoma: Adverse Clinical and Molecular Features Similar to Rhabdoid Tumors*	2009	8	USA
Al Nassan et al.	*INI1 (BAF 47) Immunohistochemistry is an Essential Diagnostic Tool for Children With Hepatic Tumors and Low Alpha Fetoprotein*	2010	2	Jordan
Machado et al.	*Immunohistochemical Study as a Tool in Differential Diagnosis of Pediatric Malignant Rhabdoid Tumor*	2010	1	Spain
Lautz et al.	*Successful Nontransplant Resection of POST-TEXT III and IV Hepatoblastoma*	2011	2	USA
Martelli et al.	*Malignant rhabdoid tumour of the liver in a seven-month-old female infant: A case report and literature review*	2013	1	USA
Agarwala et al.	*Malignant rhabdoid tumor of liver*	2014	1	India
Kachanov et al.	*Malignant rhabdoid tumor of the liver presented with initial tumor rupture*	2014	1	Russia
Marty et al.	*Tumeurs rhabdoides du nourrisson : une urgence diagnostique*	2014	1	France
Ting et al.	*A Baby Girl with Distended Abdomen and Unusual Course of Umbilical Catheter*	2014	1	Canada
Oita et al.	*Malignant rhabdoid tumor of the liver: a case report and literature review*	2015	1	Japan
Zhou et al.	*Is INI1-retained small cell undifferentiated histology in hepatoblastoma unfavorable?*	2015	7	USA
Kupeli et al.	*Infantile rhabdoid tumor mimicking hepatic hemangioendothelioma*	2016	1	Turkey
Vlajnic et al.	*Fine Needle Aspiration in the Diagnosis and Classification of Hepatoblastoma*	2016	1	France
Farber et al.	*Prognostic factors and survival in non-central nervous system rhabdoid tumors*	2017	1	USA
Cornet et al.	*Rhabdoid tumor of the liver: Report of 6 pediatric cases treated at a single institute*	2018	6	France
Kapral et al.	*Pediatric hepatic rhabdoid tumor: A rare cause of abdominal mass in children*	2018	1	USA
Fazlollahi et al.	*Malignant Rhabdoid Tumor, an Aggressive Tumor Often Misclassified as Small Cell Variant of Hepatoblastoma*	2019	6	USA
Bharti et al.	*Common and Rare Histological Variants of Hepatoblastoma in Children: A Pathological Diagnosis and Review of the Literature*	2021	1	India
Ivana et al.	*Malignant rhabdoid tumor—The great mimicker: Two case reports*	2021	2	Serbia
