# Supplementary material for: A Systematic Review and Meta-Analysis of Malignant Rhabdoid and Small Cell Undifferentiated Liver Tumors: A Rational for a Uniform Classification
Source: Cancers (Basel). 2022 Jan 6;14(2):272. doi: 10.3390/cancers14020272 (PMC8774069; doi:10.3390/cancers14020272)
Supplement: Supplementary file 1 [file cancers-14-00272-s001.zip › File S3.pdf]

Supplementary Material File S3: Risk of bias assessment of case reports/series

Study	Selection	Ascertainment	Causality	Reporting	Overall risk of bias
Gonzalez-Crussi et al.	high	high	moderate	high	high
Parham et al.	moderate	moderate	moderate	high	high
Pierro et al.	low	moderate	moderate	high	high
Hunt et al.	high	moderate	moderate	moderate	moderate
Foschini et al.	high	moderate	moderate	moderate	moderate
Hansen et al.	high	moderate	moderate	moderate	moderate
DiCori et al.	high	high	moderate	high	high
Gururangan et al.	low	moderate	moderate	moderate	moderate
Weymann et al.	high	moderate	moderate	moderate	moderate
Kaiserling et al.	moderate	moderate	moderate	high	high
Scheimberg et al.	low	moderate	moderate	high	high
Tsunoda et al.	high	moderate	moderate	high	high
Pogacnik et al.	high	moderate	moderate	moderate	moderate
Jimenez et al.	high	moderate	moderate	moderate	moderate
Kelly et al.	high	moderate	moderate	moderate	moderate
White et al.	low	moderate	moderate	high	moderate
Donner et al.	high	moderate	moderate	moderate	moderate
Ohyama et al.	high	moderate	low	moderate	moderate
Sattler et al.	high	moderate	moderate	high	high
Ravindra et al.	high	low	low	moderate	low
Weir et al.	high	moderate	moderate	high	high
Katzenstein et al.	high	moderate	moderate	moderate	moderate
Yuri et al	high	moderate	moderate	low	moderate
Kuroda et al.	high	moderate	moderate	high	high
Clairotte et al.	high	moderate	moderate	low	moderate
Philipose et al.	high	moderate	High	high	high
Jayaram et al.	high	low	low	low	low

Madigan et al.	low	moderate	moderate	high	high
Wagner et al.	high	moderate	moderate	low	moderate
Boudeaut et al.	low	moderate	moderate	high	moderate
Gutweiler et al.	high	low	moderate	moderate	moderate
Wu et al.	low	low	moderate	high	moderate
Abe et al.	high	low	low	moderate	Moderate
Trobaugh-Lotrario et al..	moderate	moderate	high	high	high
Marzano et al.	moderate	moderate	moderate	low	moderate
Al Nassan et al.	moderate	moderate	moderate	low	moderate
Machado et al.	moderate	moderate	moderate	moderate	moderate
Martelli et al.	high	moderate	moderate	moderate	moderate
Agarwala et al.	high	moderate	moderate	high	high
Kachanov et al.	high	low	low	low	low
Marty et al.	moderate	moderate	moderate	moderate	moderate
Ting et al	high	moderate	moderate	moderate	moderate
Oita et al.	high	moderate	moderate	low	moderate
Vlajnic et al.	low	moderate	high	moderate	high
Kupeli et al.	high	moderate	moderate	moderate	moderate
Farber et al.	low	moderate	high	high	high
Kapral et al.	high	low	moderate	low	low
Bharti et al.	moderate	moderate	moderate	high	high
Ivana et al.	moderate	moderate	low	low	low
