# Supplementary material for: A Systematic Review and Meta-Analysis of Malignant Rhabdoid and Small Cell Undifferentiated Liver Tumors: A Rational for a Uniform Classification
Source: Cancers (Basel). 2022 Jan 6;14(2):272. doi: 10.3390/cancers14020272 (PMC8774069; doi:10.3390/cancers14020272)
Supplement: Supplementary file 1 [file cancers-14-00272-s001.zip › File S4.pdf]

Supplementary Material File S4: ROB of retrospective observational studies with MINORS

MINORS Items	Haas et al.	Bajpai et al.	De Ioris et al	Zhou et al.	Cornet et al.	Fazlollahi et al.	Lautz et al.
Clearly stated aim	1	1	2	2	2	2	2
Inclusion of consecutive patients	2	2	2	2	2	2	2
Prospective collection of data	2	1	2	1	1	1	1
Endpoints appropriate to aim of study	1	1	2	2	2	2	2
Unbiased assessment of the study endpoint	1	1	1	1	1	1	1
Follow-up period appropriate to aim of study	2	1	2	2	1	1	2
Loss to follow up less than 5%	2	2	2	2	2	2	2
Prospective calculation of the study size	0	0	0	0	0	0	0
Total	11/16	9/16	13/16	12/16	11/16	11/16	12/16
